# Design of a case management model for people with chronic disease (Heart Failure and COPD). Phase I: modeling and identification of the main components of the intervention through their actors: patients and professionals (DELTA-icE-PRO Study)

**DOI:** 10.1186/1472-6963-10-324

**Published:** 2010-12-02

**Authors:** Jose M Morales-Asencio, Francisco J Martin-Santos, Juan C Morilla-Herrera, Magdalena Cuevas Fernández-Gallego, Miriam Celdrán-Mañas, Francisco J Navarro-Moya, Maria M Rodríguez-Salvador, Francisco J Muñoz-Ronda, Elena Gonzalo-Jiménez, Almudena Millán Carrasco

**Affiliations:** 1 Faculty of Health Sciences. University of Málaga, Málaga, Spain; 2 Healthcare District, Andalusian Healthcare Service, Málaga, Spain; 3 Andalusian School of Public Health, Granada, Spain; 4 District of Primary Health Care. Andalusian Health Service. Málaga, Spain; 5 Healthcare District, Andalusian Healthcare Service, Almería, Spain; 6 Hospital Torrecárdenas. Andalusian Healthcare Service, Almería, Spain

## Abstract

**Background:**

Chronic diseases account for nearly 60% of deaths around the world. The extent of this silent epidemic has not met determined responses in governments, policies or professionals in order to transform old Health Care Systems, configured for acute diseases. There is a large list of research about alternative models for people with chronic conditions, many of them with an advanced practice nurse as a key provider, as case management. But some methodological concerns raise, above all, the design of the intervention (intensity, frequency, components, etc).

**Methods/Design:**

*Objectives*: General: To develop the first and second phases (theorization and modeling) for designing a multifaceted case-management intervention in people with chronic conditions (COPD and heart failure) and their caregivers. Specific aims: 1) To identify key events in people living with chronic disease and their relation with the Health Care System, from their point of view. 2) To know the coping mechanisms developed by patients and their caregivers along the story with the disease. 3) To know the information processing and its utilization in their interactions with health care providers. 4) To detect potential unmet needs and the ways deployed by patients and their caregivers to resolve them. 5) To obtain a description from patients and caregivers, about their itineraries along the Health Care System, in terms of continuity, accessibility and comprehensiveness of care. 6) To build up a list of promising case-management interventions in patients with Heart Failure and COPD with this information in order to frame it into theoretical models for its reproducibility and conceptualization. 7) To undergo this list to expert judgment to assess its feasibility and pertinence in the Andalusian Health Care. *Design*: Qualitative research with two phases: For the first five objectives, a qualitative technique with biographic stories will be developed and, for the remaining objectives, an expert consensus through Delphi technique, on the possible interventions yielded from the first phase. The study will be developed in the provinces of Almería, Málaga and Granada in the Southern Spain, from patients included in the Andalusian Health Care Service database with the diagnosis of COPD or Heart Failure, with the collaboration of case manager nurses and general practitioners for the assessment of their suitability to inclusion criteria. Patients and caregivers will be interviewed in their homes or their Health Centers, with their family or their case manager nurse as mediator.

**Discussion:**

First of a series of studies intended to design a case-management service for people with heart failure and COPD, in the Andalusian Health Care System, where case management has been implemented since 2002. Accordingly with the steps of a theoretical model for complex interventions, in this study, theorization and intervention modeling phases will be developed.

## Background

Chronic diseases account for nearly 60% of deaths around the world. The extent of this silent epidemic has not met determined responses in governments, policies or professionals in order to transform old Health Care Systems, configured for acute diseases [1].

There is plenty of evidence which suggests the failure of conventional health care models faced with this scenario [2]. The consequences of a fragmented Health Care System on patients are revealed in terms of avoidable hospital admissions [3], contradictory diagnoses or information from health care providers, or else, duplicated tests and examinations [4]. So the healthcare system "transfers" on to patients the way its services are organised, with fragmented, separate care - as if each intervention for the same user were applied to different patients. It seems unavoidable the need of a more comprehensive healthcare, with high emphasis on improving the effectiveness, the continuity of patient care and the diversification of services, in a person-centered care focus.

There is a large list of research about alternative models for people with chronic conditions, most of them locally developed, with an extensive and varied range of organizational approaches, providers, devices and technologies [5]. Many of these models have a nurse with advanced roles as one of the main providers (nurse practitioners, case management, etc). This is not extraordinary as the delivery of care to chronic patients requires a flexible, case-by-case approach, adapting healthcare to the various stages in the disease, the individual's needs, his or her interests and caregivers [6]. Nurses are naturally disposed to guaranteeing most of these premises, given their deep-rooted humanistic approach, in which understanding the life-experience of the disease and experiencing dependency, along with the human responses that arise under these circumstances, steer decision-making regardless of the underlying medical process in question [7].

Case management, integrated care, disease management, nurse-led outpatient clinics, community matrons... account for a large list of modalities [8,9] in which many interventions have been deployed: screening of risk factors, multidimensional assessments, patient and caregivers education and counseling, drug adjustment, telephone follow-up, tele-care interventions, discharge planning, home visiting, clinical consultancy, and the most of them sustained on clinical pathways and evidence-based recommendations [10].

The review of the research raises a serial of methodological issues which need to be resolved. Not always the exact hierarchy of interventions that obtains the best outcomes is well-known [11]. In other cases, the models are not grounded on strong conceptual frames, despite along the past years some theoretic proposals have been reported, either from an individual viewpoint, or from a system one. In the first case, the Shifting Perspectives Model from Paterson [12] or the Illness Trajectory from Corbin and Strauss [6] highlight the value of taking into account the patients' values and perceptions along the experience of chronic disease. In the second case, the Chronic Care Model from Wagner [13,14], has been developed and implemented over more than a thousand of Health Care Organizations, and a recent meta-analysis has reported key outcomes in diabetes, asthma, depression or heart failure (HF) [15,16].

Concerning the interventions, these are complex, multifaceted and not always the exact ingredients and doses are well-known, with the consequent barriers to reproduce the results into another context. Fortunately, some approaches have been designed to undertake complex interventions with a fairly degree of reliability [17]. Further, the combination of quantitative and qualitative research methods can help manifestly to illuminate some gaps [18-21].

Our research team has been investigating from 2002 the effectiveness of alternative models for long term care in the home environment. We have reported elsewhere the benefits of a case management model implemented in the Andalusian Health Care System in home care, in terms of improvement of accessibility, care coordination, patients functionality, satisfaction and caregivers' burden [22].

But the current case management model in our region has not people with chronic conditions as a specifically defined target population. Only when they have one of the four criteria to be included into the home care program (immobilized, terminal care, hospital discharges or caregivers) and they have complex needs, they receive this intervention. Moreover, many of the people with chronic diseases are spread along the Health Care System in a myriad of isolated programs and services. Therefore, this is a perceptible area of improvement and, what is more: the Andalusian Health Care System is well positioned for this challenge, as it has a long experience with case management (from 2002), which is fully inserted into the Primary Health Care and the acute hospitals.

All these reasons led us to explore the features that our present case management system should incorporate to supply the current demand for chronic conditions, feasible in our context, making the most of the existing resources, with a close association with other initiatives deployed in our Health Care System and, finally, adapted to the needs and demands of patients and their caregivers.

Nowadays, it would be possible to design directly an experimental model with the interventions reported in the literature about case management in chronic care, but some of the methodological flaws afore mentioned warn us about doing it.

We have considered that a grounded conceptualization and modeling, prior to any experiment, would be helpful and could prevent some of the issues referred. For this purpose, we have selected the framework suggested by Campbell et al. for the design of complex interventions [23]. A process with several sequential phases is defined by this framework, which can be compared with those of a drug development.

The first step is to identify the evidence that the intervention might have the desired effect in a called "pre-clinical phase". This may come from the review of the theoretical basis, previous studies, etc. This could lead to changes in the hypothesis and improved specification of potentially active ingredients or prevent the possible effects derived from the context. After this one, a phase for defining the components of the intervention is proposed. Qualitative research can be used in this phase to show how the intervention works or to find potential barriers. Likewise, this might help to improve further experimental designs and how the components will relate among them. Descriptive studies can be developed to propose variants of the service.

Then, on next phases, exploratory studies are developed in order to describe the constant and variable ingredients of the intervention and definitive ones, intended to compare the intervention whole defined versus an adequate alternative. In a final phase, long-term evaluations in non-controlled contexts would be developed.

For our purpose, two of the main chronic patients who visit our Health Care System will be targeted: people with HF and COPD [24,25]. There is an extensive set of studies which have reported a varied catalogue of interventions in these patients, and provide a good knowledge base for the theoretical phase.

Many systematic reviews and meta-analysis have shown important effects of these interventions in HF patients on mortality, quality of life, or hospital readmissions [26,27]. In COPD patients, self-care and health promotion programs have decreased exacerbation episodes [28], and in some cases, hospital utilization [29]. In moderate COPD subgroup, home care programs have revealed fair reductions in mortality and improvements on quality of life [30]. Positive results have been reported also for hospital at home schemes [31]. Nevertheless, there are some concerns derived from the heterogeneity of interventions and studies [32]. A systematic review which evaluated the impact of the Chronic Care Model in COPD patients, reported that those studies which implemented at least two components of the model, achieved a considerable reduction in Emergencies frequentation (RR 0.58 95%CI:0.42 to 0.79) and urgent hospital readmissions and length of stay (RR 0.78 95%CI: 0.66 to 0.94). Conversely, no effects over lung function, symptoms or quality of life were detected [33].

It is very likely that many of these interventions have been arranged with a modest contribution from patients' values and preferences, and poorly adapted to the context [20,21]. Further, many studies have been performed in Health Care Systems with high incentives for cost restraints and with heterogeneous levels of leadership given to Primary Health Care.

Therefore, before setting a case management service for people with chronic conditions, from the current home care case management service, it would be reasonable to explore how they live with their illness, the key features of their relation with the Health Care System, the interactions with Health Care providers, how they cope to these situations and which resources they make use of, in order to discover non-met needs susceptible to be covered by case management. The analysis of this information can provide elements for delineating a range of interventions to be tested in further phases. Additionally, these results could be subjected to expert consensus for reviewing its feasibility and pertinence in the Andalusian Health Care context, which can be an important way of exploring potential barriers and facilitators before its implementation.

In summary, this study is the first of a series, conceptualized from the Campbell's proposal for delineating complex interventions, that will undertake the first two phases (preclinical and modeling) for the design of a case management service for people with HF and COPD and their caregivers, in the Andalusian Public Health Care System.

## Methods/Design

### Specific aims

1) To identify key events in people living with chronic disease and their relation with the Health Care System, from their point of view.

2) To know the coping mechanisms developed by patients and their caregivers along the story with the disease.

3) To know the information processing and its utilization in their interactions with Health Care providers.

4) To detect potential unmet needs and the ways deployed by patients and their caregivers to resolve them.

5) To obtain a description from patients and caregivers, about their itineraries along the Health Care System, in terms of continuity, accessibility and comprehensiveness of care.

6) To build up a list of promising case-management interventions in patients with HF and COPD with this information in order to frame it into theoretical models for its reproducibility and conceptualization.

7) To undergo this list to expert judgment to assess its feasibility and pertinence in the Andalusian Health Care

### Design

The study will have two sub-phases:

-For the first five objectives, a qualitative design based on the life story method will be performed, focused only in the period of life with the illness. Through this technique the research team will try to obtain the patients and caregivers point of view about living with the condition, from its onset and along subsequent years. In this process, whenever possible, cross narratives will be used among patients and their main caregiver. As well, any documents, materials, personal objects that give sense to their story, will be analyzed (personal records, diaries, resources used for meeting needs derived from medication, appointments, etc). With this analysis, a map of situations, needs, contexts and paths will be traced.

-For the two last objectives, a systematic review will be developed to build up a catalogue with the main case management interventions reported in the literature, which will be confronted to the information drawn from the first phase. Following, an expert consensus through Delphi technique will be used. Experts will be asked to pronounce about the feasibility and pertinence of a list of interventions and scenarios performed through a case management service, resulting from the previous research phase.

### Subjects and sample

#### Phase 1

The study will be performed in the provinces of Granada, Almeria and Malaga, in the Southern Spain, along the Primary Health Care Districts of these provinces.

Sampling: An intentional purposive sampling will be initiated from the list of patients included in the Primary Health Care Centers with the diagnosis of HF or COPD and, in addition, from readmission registers at the reference Hospitals of each Primary Health Care District. Once they have been selected, the research team will contact to their general practitioner (GP) or family nurse to know their estimation about the suitability of these ones as key informants, with the criteria described later on. The family nurse will act as mediator and will introduce the patient to the researchers.

The initial criteria for selecting patients will be gender, type of disease (COPD-respiratory insufficiency or HF) and experiences of hospital admissions due to their disease. The segmentation by gender criteria responds to the known effect of this factor in the management and relation of patients with the Health Care System in respiratory and cardiovascular chronic conditions [34,35].

Accordingly with theoretic sampling, the relation among the objectives and the information obtained will be reviewed permanently, in order to advance in the refining of the deliberated sampling.

The intentional selection will be guided by the representativeness and narrative richness. Initially, two cases from each segment of patients (COPD/HF, male/female, with/without hospital admission) will be interviewed, with the aim to obtain an initial view for the subsequent sampling. This process will be continued until the information gets saturated. Along the interviews and continuous analysis, non-confirmative cases will be selected for verifying emerging patterns.

Interviews will be performed in the patients' homes or in the Primary Health Care Centers (depending on patients' preferences), to obtain the most encouraging communication atmosphere possible.

Deep interviews will be used, with a semi-structured guide for fairly specific topics to be covered. These topics are based on testimonies reported on Health Talk Online web site (http://www.healthtalkonline.org/heart_disease/Heart_Failure) and have been discussed among the research team. These topics only states the starting point, but flexibility will be present along the course of the interview and whatever additional issues that could be raised along the story will be assumed, due to the discursive nature of the technique.

Taking into account the patients characteristics, interviews will take approximately one hour, for avoiding informants' exhaustion. If necessary, two or more sessions will be scheduled until completing the narration.

Interviewers will annotate any circumstances, facts or elements of interest along the interview that could help to the relativization of findings during the analysis.

All the interviews will be digitally recorded with PC software and a verbatim transcript for further analysis with ATLAS Ti 5.6 software will be prepared for a person unrelated to the research team. Along this process, all the data will be anonymized to guarantee confidentiality.

### Analysis

A first reading for discovering emerging themes will be performed. Following, a coding scheme will be created from these emerging themes. The codes will be reviewed by the research team in order to get consensus about their validity for the analysis. All the interviews will be codified and the codes will be undergone to continuous refinement by the researchers throughout the data analysis. Once coding process will be finished, the non-coded paragraphs will be rereaded to identify new significant passages in order to assign a code to them. Codes will be grouped in code-families with ATLAS Ti, for data reduction and identifying relations among them. Through the ATLAS Ti Network Manager Tool, conceptual maps with the main results will be built up. Finally, data will be interpreted along the context they were collected, looking for possible influences of researchers in the patients' discourse.

### Credibility and limitations

Factors as patients' expectations, their lack of confidence to researchers, exhaustion, or recall shortcomings, can limit the credibility of the results. In order to avoid these potential limitations, the following actions will be taken:

- Expectations control: Subjects can build expectations about the study and treat to adapt their narrative to satisfy them. To counteract this possible phenomenon, written and verbal information about the study aims will be provided to the participants prior to the interviews. The patients' family nurse will anticipate the purpose and features of the interview and they will introduce the researchers. It will be highly remarked to the patients the importance of an open discourse, and that this will not affect at all to their usual health care.

-Researchers will take special care on watching for patients' exhaustion signs. In this case, interviews will be deferred for another moment.

-Confidentiality: the exclusive use of the data for research purposes and the permanent anonymity of them will be guaranteed to the patients. Researchers will explain the need of audio recording the interviews and the possibility of refusal if they do not agree with this procedure.

-Cross narrative controls: the interviews will be held in presence of their main caregiver in order to enhance possible shortcomings in their recalls. If the caregivers' presence is not possible, additional interviews with them will be scheduled if necessary.

-Coding process: the review of the whole codes by all the research team will assure the rigor in this process.

#### Phase 2

With the information drawn from the first phase, a catalog with potential ingredients of a comprehensive case management model will be made up. For this purpose, usual components will be taken from the literature and classified accordingly with the Taxonomy of the American Heart Association for Disease Management [36] and the Chronic Care Model [13]. The interventions will be extracted through a systematic review about case management and disease management studies with the criteria specified in Table 1 and placed along the elements of the taxonomy and model cited previously. The research team will confront the needs reported by the patients in phase 1 with the interventions selected, in order to relate them in multiple directions. In the case of any problem or need detected with no intervention available, the research team will propose a description of possible actions, taking into account the context of the Andalusian Health Care.

**Table 1:** Criteria for the systematic review about interventions on case management.

Type of studies:	Randomized controlled trials and systematic reviews.
Type of participants:	Adult patients with HF or COPD

Type of interventions:	Diseases management programs or case management services where the role is performed by nurses, and is targeted to HF or COPD patients, with or without use of communication technologies for telecare.

Outcomes considered:	Physical or psychosocial health outcomes, satisfaction, measures of care delivery and use, and costs.

Data collection and analysis:	eligibility, assessment of the quality of studies and data extraction will be performed by two reviewers independently. RevMan 5 will be used to organize the revision.

Search strategy:	Searches will be performed in MEDLINE, CINAHL, EMBASE, COCHRANE, WEB OF SCIENCE, SCOPUS, DARE, HEN, EUROSCAN, BIBLIOTECA VIRTUAL DE SALUD, CUIDEN, IME.

Languages:	Spanish and English.

Once the list will be made up, an expert panel for the Delphi technique will be delineated with the following criteria:

1) To be a health professional related to the care of people with COPD or HF, and

2) To have a thorough knowledge on case management, disease management or chronic care models and,

3) To have a thorough knowledge about the Andalusian Health Care System.

With these criteria, 50 panelists will be chosen among these profiles:

Nurse case managers (12), General practitioners (4), Family nurses (4), Cardiologists (3), Pneumologists (3), General internists (3), Cardiovascular nurses (4), Respiratory care nurses (4), Emergency Care nurses (2), Internal medicine nurses (4), Primary Health Care Executives (2), Hospital Executives (2) and representatives from the Spanish Chronic Patients Coalition (3).

They will be invited to participate through a Delphi technique with several rounds. In the first one, all the interventions and proposals from the first phase will be included and through a Likert scale (range 1-9) they will have to state their judgment about two dimensions: the feasibility and the pertinence of the interventions along the Andalusian Health Care System.

The voting system will be conducted through a web site, with restricted access and automated data quality control, in order to reduce systematic errors due to an inadequate data introduction. The responses from the first round will be concealed to the panelists until they finish it. Following this phase, data will be processed and the panel will receive feed-back about the results, before initiating a second round with all the items without consensus. This process will be repeated sequentially until full agreement will be ascertained.

### Analysis

Descriptive and spread statistics will be applied to Delphi results. The consensus criteria will include percentile and interquartile range.

### Ethical approval

The study was revised and approved by the Ethics and Research Committee of the Andalusian School of Public Health. Informed consent will be elicited from all the participants.

## Discussion

This is the first of a series of studies intended to design a case-management service for people with heart failure and COPD, in the Andalusian Health Care System. Accordingly with the steps of a theoretical model for complex interventions, in this study, theorization and intervention modeling phases will be developed.

A past history of success in case management implementation along the Andalusian Health Care System for people with home care needs, does not guarantee that a mere extension of services for people with chronic conditions will achieve in the same direction. Despite many of the home care population suffers chronic conditions; the nature and scope of the service are not explicitly intended to this target.

Moreover, difficulties have been reported when reproducing some complex interventions in different contexts. So, we have considered that a previous conceptualization and modeling would be helpful for policy-makers in order to define the service, to identify potential barriers and to guide the implementation process along the Health Care System. Furthermore, it would contribute to detect those interventions that case managers are already providing and that would require a minimal support and upgrade for them.

## List of abbreviations used

COPD: Chronic Obstructive Pulmonary Disease; HF: Heart Failure; RR: Relative risk.

## Competing interests

The funding organization had no role in the study design or the writing and publication of this article. The authors declare that they have no competing interests.

## Authors' contributions

JMMA participated in the conception and design of the study and drafted the first version of it, as well as the manuscript. FJMS, JCMH, MCFG, MCM, FJNM, MMRS, FJMR, EGJ, AMC participated in the conception and design of the study, and also revised critically the draft of the manuscript, with a key intellectual contribution to the final version. All authors read and approved the final manuscript.

## Funding

This research was carried out with the support of one research grant, awarded by the Regional Health Ministry of Andalusia (Exp. 0222/2008)

## Pre-publication history

The pre-publication history for this paper can be accessed here:

http://www.biomedcentral.com/1472-6963/10/324/prepub
